# Antiemetic activity of volatile oil from *Mentha spicata* and *Mentha* × piperita in chemotherapy-induced nausea and vomiting

**DOI:** 10.3332/ecancer.2013.290

**Published:** 2013-01-31

**Authors:** Z Tayarani-Najaran, E Talasaz-Firoozi, R Nasiri, N Jalali, MK Hassanzadeh

**Affiliations:** 1 Department of Pharmacodynamics and Toxicology, School of Pharmacy, Mashhad University of Medical Sciences, Mashhad 91775-1365, Iran; 2 El Camino Hospital, 2500 Hospital Drive, Mountain View, CA 94040, USA; 3 Research Center of Natural Products Safety and Medicinal Plants, North Khorasan University of Medical Sciences, Bojnurd, 01830-49504, Iran; 4 Pharmaceutical Sciences Research Center, Department of Medicinal Chemistry, School of Pharmacy, Mashhad University of Medical Sciences, Mashhad 91775-1365, Iran

**Keywords:** chemotherapy-induced nausea and vomiting (CINV), essential oils, Mentha spicata, Mentha × piperita, Lamiaceae

## Abstract

**Background:**

This study is aimed at determining the efficacy of *Mentha spicata (M. spicata) *and *Mentha *× *piperita (M. × piperita)* in preventing chemotherapy-induced nausea and vomiting (CINV).

**Methods:**

This was a randomised, double-blind clinical trial study. Prior to the study, patients were randomly assigned into four groups to receive *M. spicata* or *M*. × *piperita*. Statistical analysis included the *χ*^2^ test, relative risk, and Student’s t-test. Fifty courses were analysed for each group that met our eligibility criteria. The treatment and placebo groups applied essential oils of *M. spicata, M.* × *piperita*, or a placebo, while the control group continued with their previous antiemetic regimen. Patients or guardians recorded the number of emetic events, the intensity of nausea over 20 h of chemotherapy, as well as any possible adverse effects that occurred during this time.

**Results:**

There was a significant reduction in the intensity and number of emetic events in the first 24 h with *M. spicata* and *M. × piperita* in both treatment groups (p < 0.05) when compared with the control and no adverse effects were reported. The cost of treatment was also reduced when essential oils were used.

**Conclusion:**

*M. spicata* or *M*. × *piperita* essential oils are safe and effective for antiemetic treatment in patients, as well as being cost effective.

## Introduction

CINV is a major problem for cancer patients [1]. Approximately 70–80% of all cancer patients receiving chemotherapy, experience nausea and vomiting [2]. Despite advances in antiemetic drugs, side effects continue to be clinically significant. The management of vomiting seems to be improved at the expense of nausea, as has been indicated in reports over the years. Nausea continues to be ranked first on the list of troublesome and distressing symptoms experienced by patients despite the use of 5-HT3 receptor antagonists, and it has profound effects on the quality of life of patients [3].

Aromatic plants were used in ancient times for their preservative and medicinal properties, as well as to impart aroma and flavour to food. Hippocrates, who is sometimes referred to as the ‘father of medicine’ prescribed perfumed fumigations [4], and aromatherapy, which is the inhaled use of essential oils used for therapeutic or medical purposes. Nowadays, herbs are commonly used for cancer symptoms management treatment in adults. Spearmint *(Mentha spicata)*, and peppermint *(Mentha × piperita)* are recommended for their antiemetic and antispasmodic effects on the gastric lining and colon. Several studies show the efficacy of peppermint in reducing postoperative nausea and vomiting [5, 6].

This study evaluates the efficacy and side effects of the volatile oils from the *M. spicata* and *M. × piperita* via the oral route for the prevention of CINV in cancer patients.

## Patients and Methods

### Identification of Oil Components

Analytical gas chromatography (GC) was carried out using a 3400 varian-star cx GC chromatograph with capillary column DB-5 (30 m × 0.32 mm i.d., 0.32-μm film thickness), a He carrier gas, split ratio of 1:10, and a flame ionisation detector.

The column temperature was programmed at 60°C for one minute and then heated to 240°C at a rate of 3°C/min, then kept constant at 265°C for 20 min. GC–mass spectrometry (MS) was performed on a Thermoquest 2000 with a quadrupole detector on a capillary column DB-5 (see GC), with a He carrier gas, a flow rate of 1.5 ml/min, and an oven temperature of 265°C. The mass spectrometer was operated at 70 eV ionisation energy. Retention indices were calculated by using retention times of *n*-alkanes, which were injected after the oil, at similar chromatographic conditions. The compounds were identified by comparing retention indices (RRI and DB-5) with those reported in the literature and by comparing their mass spectra with the Wiley library or with published mass spectra (Tables 1 and 2).

### Eligibility Criteria

#### Patient characteristics

This was a randomised, double-blind clinical trial study. This study was conducted at the Omid Chemotherapy Hospital of the Mashhad Medical University in Mashhad, Iran. The inclusion criteria were patients with any cancer diagnosis (colon adeno-carcinoma, breast cancer, colorectal cancer, oesophageal cancer, liver cancer, lung cancer, Hodgkins lymphoma, non-Hodgkins lymphoma, melanoma, nasopharyngeal cancer, osteosarcoma, ovarian cancer, pancreatic cancer, sarcoma, stomach cancer, testicular cancer, and vaginal cancer), who were about to receive chemotherapy on an outpatient basis, and who were chemotherapy naïve. After the patients (or their guardians) signed informational consent forms, the patients received chemotherapy drugs (etoposide, ifosfamide, cisplatin, carboplatin, epirubicin, cyclophosphamide, adriamycin and/or irinotecan). Patients who were experiencing nausea and vomiting from causes other than chemotherapy reasons (i.e. intestinal obstruction, stomach cancer, pre-menstrual syndrome, motion sickness), or were deemed physically incapable of participating by the investigators, were excluded from the study.

The study was approved by the Ethics Committee of the Mashhad University of Medical Sciences. Identified variables for patients included age, sex, nutritional status (body mass index [BMI]), diagnosis, chemotherapy regime, previous exposure to chemotherapy, prescribed antiemetic medication and nausea or vomiting in previous treatments.

Prior to each cycle, patients underwent a physical examination and a complete haematological and urinary evaluation to assure optimal conditions to receive treatment.

#### Treatment plan

During each cycle, patients received their normal antiemetic regimen (granisetron, dexamethason or metocloprimide) plus spearmint and peppermint capsules (containing two drops of each essential oil and filled with sugar) every four h. This was administered 30 minutes before the patients received their chemotherapy treatment, again four h after the first capsule and finally, four h later at home.

Patients or guardians received a self-applicable questionnaire to record the number of emetic events and the intensity of the nausea during the next 24 h period following chemotherapy.

Patients also underwent a complete physical evaluation on each day of their hospitalisation in order to identify any abnormal event. Evaluation of these results was performed by a blind investigator.

#### Statistical analysis

Statistical analysis included the Mann–Whitney U test or the Kruskal–Wallis test to compare the median value of emetic events for each day in both groups, as the assumptions for Student’s t-test or ANOVA were not fulfilled. Risk analysis included a relative risk analysis, which was performed by comparing the incidence of the effect (presence of CINV in both groups. Pearson’s χ^2^ statistic was used to determine the association of nominal or ordinal variables. The Fisher’s exact test was performed when conditions were not met.

## Results

The volatile oil from the *M. spicata* and *M. × piperita* were examined by GC and GC–MS. Thirteen compounds were identified in the essential oils of *M. × piperita* and 14 compounds in *M. spicata,* which were then evaluated for their preventive properties in CINV (Tables 1 and 2).

Fifty patients were analysed for each group. Clinical details of the patients are summarised in Table 3. No differences were found between treatment groups except for a greater proportion of normal weight in each group (Table 3).

Efficacy was assessed based on the number of emetic episodes (vomiting and retching), and intensity and duration of nausea.

Intensity of nausea was scored between 0 and 100: absent (0) severe (100) (Table 4).

Patients treated with *M. spicata* and peppermint were found to have a statistically significant reduction of the median emetic events during 24 h of treatment (acute phase) in which patients treated with placebo presented up to 1.8 emetic events versus 0.6 and 0.7 in the *M. spicata* and *M. × piperita*, respectively (p < 0.05) (Table 5).

The intensity of nausea was also significantly reduced in the *M. spicata* and *M. × piperita* groups during 24 h of treatment compared with placebo (p < 0.05) (Table 5).

There was no statistical difference between spearmint and peppermint in controlling emetic events and intensity of nausea (p > 0.05).

None of the patients reported or presented any adverse effect during treatment with *M. spicata* and *M. × piperita*.

The cost of treatment with *M. spicata* and *M. × piperita* was significantly lower than with granisetron.

## Discussion

Nausea and vomiting are still an obvious problem in patients receiving chemotherapy. On conditioning with more than 24 h of chemotherapy, patients are at risk for CINV throughout the entire treatment period. Aromatherapy is the inhaled use of essential oils for therapeutic or medical purposes. Ginger *(Zingiber officinale)*, spearmint (*M. spicata*), and peppermint (*M. × piperita*) are recommended for their antiemetic and antispasmodic effects on the gastric lining and colon. Several studies show the efficacy of peppermint in reducing postoperative nausea and vomiting [6], chemotherapy-induced nausea [7], and colonic spasms during colonoscopy [8, 9] and after colostomy surgery [10].

Exposure to ionising radiation induces nausea and vomiting, such as in radiation accidents. Hence, plants with antiemetic activity (e.g. *Centella asiatica, M. × piperita, and Zingiber officinale)* have been evaluated for their ability to provide radiation protection [11–14]. Nevertheless, *M. × piperita* and *M. spicata* essential oils have not been fully analysed in populations.

In this study, we used spearmint and peppermint capsules (containing two drops of each essential oil every four h, filled with sugar, and administered 30 minutes before they received their chemotherapy treatment, again four h after first capsule and finally four h later in home) for all patients, independent of their age or weight. Furthermore, no significant side effects were found when increasing the dose.

Although it was not our aim to compare spearmint and peppermint, we found no difference between these two herbs in the control of vomiting in the first 24 h (p > 0.05).

Based on our results, we have concluded that the essential oils of spearmint and peppermint are less expensive, and a safe and effective therapeutic option for the treatment of chemotherapy-induced nausea and emesis in patients.

## Figures and Tables

**Table 1.** GC–MS data of the components of the essential oils of *M. × piperita*

Compounds	Retention time (min)	Kovat’s retention index	(%)
**Limonene**	481	1040	**5.96**
Menthone	761	1146	1.12
Borneol	789	1156	0.68
Terpinen-4-ol	820	1166	0.99
***cis***-**Dihydrocarvone**	866	1186	**19.19**
*trans*-Dihydrocarvone	885	1191	1.06
**Pulegone**	973	1229	**13.30**
**Carvone**	984	1238	**42.53**
Piperitone	1011	1245	1.52
*α*-Terpinenyl acetate	1264	1323	3.45
*β*-Carvyl acetate	1296	1353	1.06
*β*-Bourbonene	1355	1370	1.46
***β***-**Caryophyllene**	1442	1397	**6.78**
*α*-Humulene	1527	1435	0.88

**Table 2.** GC–MS data of the components of the essential oils of *M. spicata*

Compounds	Retention time (min)	Kovat’s Retention index	Composition (%)
α-Pinene	319	934	0.82
Sabinene	397	989	2.39
**Limonene**	481	1038	**6.35**
1,8-Cineole	485	1039	2.29
Menthone	761	1144	1.75
**Isomenthone**	785	1155	**5.75**
**α-Terpineol**	852	1185	**8.91**
Carveol	923	1212	0.54
**Pulegone**	973	1240	**56.28**
Piperitone	1011	1252	2.29
Thymol	1113	1289	4.51
Carvacrol	1137	1294	2.63
**Longifolene**	1404	1397	**5.47**

**Table 3.** Characteristics of patients

	Control	Placebo	*M. × piperita*	*M. spicata*	p value^*^
**Age**	**43±11**	**44±15**	**46±15**	**42±15**	0.056
≤50	33 (66%)	34 (68%)	40 (80%)	31 (62%)	
>50	17 (34%)	16 (32%)	10 (20%)	19 (38%)	
**Sex**					0.274
Male	16 (32%)	14 (28%)	14 (28%)	22 (44%)	
Female	34 (68%)	36 (72%)	36 (72%)	28 (56%)	
**History of TM use**					0.203
Yes	26 (52%)	21 (42%)	26 (52%)	17 (34%)	
No	24 (48%)	29 (58%)	24 (48%)	33 (66%)	
**Nutritional status**					0.004
Underweight	12 (24%)	13 (26%)	5 (10%)	3 (6%)	
Normal	37 (74%)	30 (60%)	41 (82%)	37 (74%)	
Obese	1 (2%)	7 (14%)	4 (8%)	10 (20%)	
**Antiemetic treatment with chemotherapy**					
Granistron	29 (58%)	37 (74%)	29 (58%)	34 (68%)	0.253
Dexamethasone or metoclopramide	21 (42%)	13 (26%)	21 (42%)	16 (32%)	
History of nausea in previous chemotherapy	32 (64%)	25 (50%)	29 (58%)	33 (66%)	0.36
History of vomiting in previous chemotherapy	16 (32%)	12 (24%)	13 (26%)	19 (38%)	0.414

* Pearson’s χ^2^ statistic or Fisher’s exact test.

**Table 4.** Grades of nausea and vomiting

	Grade 1	Grade 2	Grade 3	Grade 4
Nause^a^	Absent (scored 0)	mild	moderate	Severe (scored 100)
Vomiting	Absent 1 episode/24 h	>3 episodes/24 h	3-5 episodes/24 h	>5 episodes/24 h

a Grading according to Reference [14].

**Table 5.** Summary of efficacy results (N = 50 in each group)

	Control	Placebo	*M. × piperita*	*M. spicata*	
					p value*
Retching episodes	3.1 ± 1.3	7.2 ± 2.3	2.4 ± 2.4	3.8 ± 1.2	<0.0001
intensity of nausea	52.8 ± 20.7	14.6 ± 9.1	42.1 ± 22.7	43.9 ± 12.4	<0.0001
Vomiting episodes	1.1 ± 0.4	1.8 ± 0.9	0.7 ± 0.9	0.6 ± 0.3	<0.0001
